# Automatic Segmentation of Myocardium from Black-Blood MR Images Using Entropy and Local Neighborhood Information

**DOI:** 10.1371/journal.pone.0120018

**Published:** 2015-03-26

**Authors:** Qian Zheng, Zhentai Lu, Minghui Zhang, Lin Xu, Huan Ma, Shengli Song, Qianjin Feng, Yanqiu Feng, Wufan Chen, Taigang He

**Affiliations:** 1 Zhengzhou University of Light Industry, Zhengzhou 450002, China; 2 School of Biomedical Engineering, Southern Medical University, Guangzhou 510515, China; 3 University of Electronic Science and Technology of China, Chengdu 611731, China; 4 Cardiovascular Sciences Research Centre, St George’s, University of London, London SW17 0RE, United Kingdom; 5 Biomedical Research Unit, Royal Brompton Hospital and Imperial College London, London SW7 2AZ, United Kingdom; Shenzhen institutes of advanced technology, CHINA

## Abstract

By using entropy and local neighborhood information, we present in this study a robust adaptive Gaussian regularizing Chan–Vese (CV) model to segment the myocardium from magnetic resonance images with intensity inhomogeneity. By utilizing the circular Hough transformation (CHT) our model is able to detect epicardial and endocardial contours of the left ventricle (LV) as circles automatically, and the circles are used as the initialization. In the cost functional of our model, the interior and exterior energies are weighted by the entropy to improve the robustness of the evolving curve. Local neighborhood information is used to evolve the level set function to reduce the impact of the heterogeneity inside the regions and to improve the segmentation accuracy. An adaptive window is utilized to reduce the sensitivity to initialization. The Gaussian kernel is used to regularize the level set function, which can not only ensure the smoothness and stability of the level set function, but also eliminate the traditional Euclidean length term and re-initialization. Extensive validation of the proposed method on patient data demonstrates its superior performance over other state-of-the-art methods.

## Introduction

According to the World Health Organization, an estimated 17.5 million people died from cardiovascular diseases in 2005, representing 30% of all global deaths [1]. Being able to provide an early diagnosis and treatment will dramatically reduce this death toll. Recent advances in novel imaging and computing technology and their introduction into clinical routine have shown tremendous potential towards achieving such an ambitious goal. Over the diverse range of imaging modalities, cardiovascular magnetic resonance (CMR) imaging is a unique technique that is ionizing radiation free and can provide clear anatomy of the heart. Early iron detection using myocardial T2* values derived from CMR was developed and validated in large patient populations [2]. Extracting the anatomical information of the cardiac is the fundamental step for the development of clinical applications, and obtaining reproducible and unbiased quantitative measurement of the anatomy is indeed of central importance for the success of these applications.

Manual delineation in all the images of a subject is prohibitively a labor intensive, tedious and time-consuming task. Automatic or semi-automatic algorithms are therefore highly desired. Several approaches have been reported in the literatures for medical image segmentation. These approaches can be roughly categorized into three groups, namely, clustering algorithms [3,4,5], Markov random field (MRF) methods [6,7,8], and active contour-based methods [9,10,11,12,13,14]. Clustering methods iterate between segmenting the image and characterizing the properties of each class. Three commonly used clustering algorithms are the k-means [3], fuzzy c-means algorithm [4] and the expectation- maximization (EM) algorithm [5]. Without incorporating spatial information, these methods are sensitive to noise and image intensity inhomogeneity. MRF methods [6] incorporate spatial information using the Bayesian model, which is robust to noise. The segmentation is obtained using iterative methods such as iterated conditional modes [15] or simulated annealing [16]. However, a difficulty associated with MRF models is proper selection of the parameters controlling the strength of spatial interactions. Active contour models (ACMs) have been widely used for automatic image segmentation and object tracking [17,18]. The basic idea of the ACM is to evolve a curve to fit the desired object boundary according to predefined energy function [19,20]. According to the type of adopted image features, the existing active contour models can be broadly divided into edge-driven models [9,10,11] and region-driven models [12,13,14].

For edge-driven models, energy function is constructed using edge-based image features, i.e., local intensity gradients, outputs of edge indicator, and so on. This type of methods can be applied to images with intensity in homogeneities. However, these models are not only very sensitive to image noise, but also difficult to detect weak boundaries. Moreover, the segmentation result is highly dependent on the initial contour placement.

Alternatively, region-driven models depend on the region-based image features, i.e., some statistics of the primary image features in a region. These approaches have better performance in images with weak object boundaries due to the independence on image gradients. Chan–Vese (CV) model is a classical region-based active contour model [13]. Utilizing the global information of the image, the CV model can extract the contour that has an unobvious change of gradient in the image. Therefore, the CV model can obtain a good result even if the image has weak object boundaries. Furthermore, the CV model can reserve the topological structure in the evolutionary process automatically. However, image intensity inhomogeneity often occurs in CMR images, and the CV model cannot accurately detect the boundary of the object in such images.

To segment images with image intensity heterogeneity, Li *et al*. proposed the local binary fitting (LBF) model that is able to utilize image information in local regions [21]. A kernel function was introduced to define a local binary fitting energy in a variation formulation, and the local image intensity information can be embedded into the region-based active contour model. The LBF model has better performance than the CV model in segmentation accuracy. Nevertheless, the LBF model is susceptible to the initial contour placement and configuration of controlling parameters due to the limitation of the localized energy.

Shawn Lonkton and Allen Tannenbaum presented a novel framework based on the localizing region-based active contours to segment objects with heterogeneous feature profiles [22]. This method takes the local image information into account, and has significant improvement in accuracy for segmenting heterogeneous images. The limitations of this model are its sensitivity to the initialization parameters and the window size of the local region. Wang *et al*. proposed a new local Chan-Vese model for image segmentation [23], which is based on the techniques of curve evolution, local statistical function and level set method. The limitation of this model is its sensitivity to the window size of the local region.

The black-blood T2* has been shown to be effective by yielding high contrast images, providing superior myocardial border definition, and largely reducing blood signal contamination from the myocardium [24]. In recent years, a number of left ventricular (LV) segmentation methods from CMR cine images have been proposed [25,26,27]. However, there are limited reports on LV segmentation from the black-blood double inversion recovery images. We present a robust adaptive Gaussian regularizing CV model using the entropy and local neighborhood information for automatic LV segmentation from cardiac MR images, namely SMLV (LV segmentation method) for brevity. First, the circular Hough transformation (CHT) is used to locate the epicardial and endocardial contours of LV as the initialization. Second, an adaptive window is utilized to reduce the sensitivity to initialization rather than a fixed window using a fixed neighborhood window size in the previous works [28,29]. The interior and exterior energies are weighted by the entropies of interior and exterior regions, so that the homogeneity proportions of the interior and exterior regions are adjusted adaptively, reducing the effect of the optimal configuration of controlling parameters. Third, local region energies were computed over neighborhoods of points close to the curve. To improve the local neighborhood information (LNI), an adaptive window is used to reduce the sensitivity to initialization. Fourth, the Gaussian regularization is used to not only keep the level set function smooth and stability, but also remove the traditional Euclidean length term and re-initialization.

Qualitative and quantitative evaluations of the proposed method were carried out on both synthetic images and real medical images of clinical patients in terms of accuracy and robustness. The results show that the present method can segment the LV from cardiac images accurately and efficiently.

The remaining of the paper is organized as follows: “Material and methods” describes the image data, CV model, the classic CHT and the present method in details. The experiments setup and related evaluation are given in “Results and Discussion”. Finally, some concluding remarks are included in “Conclusions”.

## Materials and Methods

### 1. Image Data

All CMR were performed at a 1.5T scanner (Siemens Sonata, Erlangen, Germany) with a four-element cardiac phased-array coil and standard electrocardiogram gating and were collected from 2006 to 2009. The final data set comprised of 111 × 8 images of 111 patients (57 male /54 female). The patients’ ages ranged from 11 to 51 years old. A single short-axis midventricular slice positioned halfway between the base and the apex of the LV was obtained by using a black-blood multi-echo gradient-echo sequence (flip angle 20°, sampling bandwidth 810 Hz/pixel, voxel size 1.56 × 1.56 × 10 mm^3^ and variable field of view (20∼30) × 40cm^2^ depending on patient size). The short-axis images were acquired at 8 echo times (TE) from 2.54ms to 17.90ms with an increment of approximately 2ms in a single breath-hold. The repetition time between radiofrequency pulses was 20ms. Black-blood sequence images were acquired in diastole after a double inversion recovery preparation pulse. For quantitative analysis of the algorithm, we compared the automatic segmentation results to the Gold Standard, which is defined by the medical experts using the computer-aided software. The study was approved by the local research ethics committee and the subjects gave their written informed consent.

### 2. Methodology

#### 2.1 Circular Hough Transformation (CHT)

**Fig 1:**
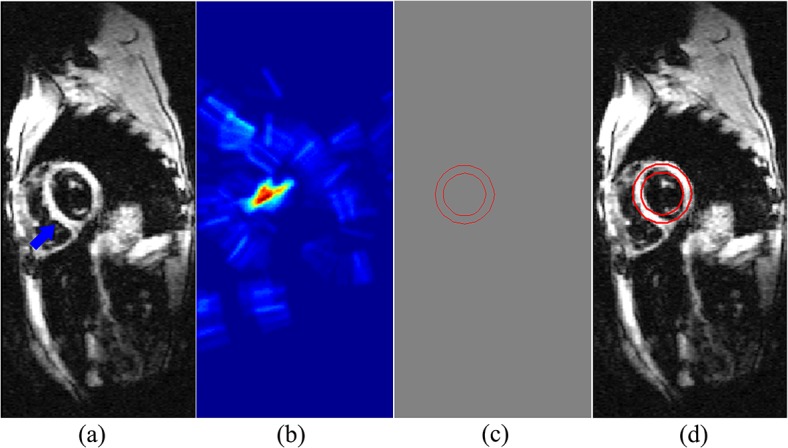
A CHT example. (a) Original image. (b) The space of CHT. (c) Detected circles. (d) The circles imposed on the image.

Proposed by Duda et al. [30], the CHT is one of the modified versions of the Hough transformation. The CHT aims to locate circular patterns in images. It is used to transform a set of feature points in the image space into a set of accumulated votes in a parameter space. Then, for each feature point, votes are accumulated in an accumulator array for all parameter combinations. The array elements that contain the highest number of votes indicate the presence of the shape. The radius and the center of the circle can also be computed by the CHT. The example of CHT is shown in **Fig. 1**. In this study, the detected circles are utilized as the initialization in order to reduce the time of the curve evolved to the object and to improve the robustness.

#### 2.2 Chan–Vese model (CV model)

Let *I* denotes a given image defined on domain Ω and let *C* be a closed curve dividing the image into object and background. The object denoted by Ω_*i*_ is the interior of the curve, and the background denoted by Ω_*o*_ is the exterior of the curve. The energy functional is given by

$$\varepsilon \left(C,c_{i},c_{o}\right)=\mu L\left(C\right)+\nu S\left(C\right)+\lambda_{in}\int_{inside\left(C\right)}{\left|I\left(z\right)-c_{i}\right|}^{2}dz+\lambda_{out}\int_{outside\left(C\right)}{\left|I\left(z\right)-c_{o}\right|}^{2}dz$$ (1)

Where *μ* is the coefficient of the length *L*(*C*) of curve *C*, *ν* denotes the coefficient of the area *S*(*C*) of Ω_*i*_, and *z* is the spatial variable; *c*_*i*_ and *c*_*o*_ stand for the means inside and outside *C*, respectively; *λ*_*in*_ and *λ*_*out*_ represent the weights of the interior and exterior of the contour *C*; The boundary of the object is obviously the minimum of *ε*(*C*,*c*_*i*_,*c*_*o*_), which can be solved using the level set method [31].

In the level set method, *C* is considered as the zero level set of a signed distance function *ϕ*, i.e., *C* = {*z*|*ϕ*(*z*) = 0}, *ϕ*(Ω_*i*_) > 0 and *ϕ*(Ω_*o*_) < 0. The energy function expressed by level set function *ϕ* is

$$\varepsilon \left(\phi ,c_{i},c_{o}\right)=\mu \int_{\Omega }\delta \left(\phi \left(z\right)\right)\left|\nabla \phi \left(z\right)\right|dz+\nu \int_{\Omega }H\left(\phi \left(z\right)\right)dz+\int_{\Omega }F\left(\phi \left(z\right)\right)dz$$ (2)

where *H*(*z*) denotes the Heaviside function and δ(*z*) is the Dirac function.

*F*(*ϕ*(*z*)) is the fitting term of the energy functional as follows

$$F\left(\phi \left(z\right)\right)=\lambda_{in}{\left|I\left(z\right)-c_{i}\right|}^{2}H\left(\phi \left(z\right)\right)+\lambda_{out}{\left|I\left(z\right)-c_{o}\right|}^{2}\left(1-H\left(\phi \left(z\right)\right)\right)$$ (3)

Minimizing the above energy function by using the steepest descent method, we obtain the following variational formulation

$$\left\{ \begin{array}{l} c_{i}=\frac{\int_{\Omega }I\left(z\right)H\left(\phi \right)dz}{\int_{\Omega }H\left(\phi \right)dz},c_{o}=\frac{\int_{\Omega }I\left(z\right)\left(1-H\left(\phi \right)\right)dz}{\int_{\Omega }\left(1-H\left(\phi \right)\right)dz}\\ \frac{\partial \phi }{\partial t}=\delta \left(\phi \right)\left[\mu div\left(\frac{\phi }{\left|\nabla \phi \right|}\right)-\nu -\lambda_{in}{\left(I\left(z\right)-c_{i}\right)}^{2}+\lambda_{out}{\left(I\left(z\right)-c_{o}\right)}^{2}\right] \end{array}\right.$$ (4)

Where *div*(∙)is the divergence.

From the energy functional of CV model, the evolution of the curve is influenced by three terms. The first two terms regularizes the contour to keep the contour smooth during the evolution. The last term in equation (2) is called the data term, which has influence on the evolution of the contour. This model does not use the edge factor to stop the evolving curve on the boundary of the object, and is based on the assumption that the interior and exterior of the curve simultaneously are statistically homogeneous. The advantage of this model is robust to noise. As is well known to all, it is very suitable for segment images with two regions that have a distinct mean of pixel intensity. The performance of the segmentation is subject to optimal configuration of controlling parameters. Moreover, this model often fails to segment the objects with image intensity inhomogeneity and complex background [21]. The above deficiencies motive us to present a new method.

#### 2.3 Entropy

Entropy is a measure of disorder, or more precisely unpredictability, which is an important concept of information theory. The entropy and its several modified versions [32,33] have been recognized as robust techniques for image segmentation. The more homogeneous of the region is, the smaller the entropy of the region. The definition of the entropy is

$$E=-\int_{\Omega }p\left(x\right)\log p\left(x\right)dx$$ (5)

where *p*(*x*) represents the probability density functions of the variable the *x* (e.g., the feature such as brightness, color, gradient). It is convenient to estimate the entropy of the interior region within the given image, *E*_*in*_(*C*) is defined by

$$E_{in}\left(C\right)=-\int_{inside\left(C\right)}p\left(I\left(z\right)\right)\log p\left(I\left(z\right)\right)dz$$ (6)

Similarly, the entropy of the exterior *E*_*out*_ is given as

$$E_{out}\left(C\right)=-\int_{outside\left(C\right)}p\left(I\left(z\right)\right)\log p\left(I\left(z\right)\right)dz$$ (7)

For the level set formulation of the CV model, we replace the unknown variable *C* by the unknown variable *ϕ*. Using the Heaviside and Dirac functions, we express the equations (6) and (7) in the following way, respectively:

$$E_{in}\left(\phi \right)=-\int_{\Omega }H\left(\phi \left(z\right)\right)p\left(I\left(z\right)\right)\log p\left(I\left(z\right)\right)dz$$ (8)

$$E_{out}\left(\phi \right)=-\int_{\Omega }\left(1-H\left(\phi \left(z\right)\right)\right)p\left(I\left(z\right)\right)\log p\left(I\left(z\right)\right)dz$$ (9)

#### 2.4 Entropy weighted CV model (ECV)

In the CV model, *μ* and ν are weight that represent the smoothness and tautness degrees of the curve, respectively. *λ*_*in*_ and *λ*_*out*_ are the weight of the homogeneities for the interior and exterior of the curve, respectively. The weight parameter is difficult to set for segmentation of heterogeneous images. CV model does not provide the strategy that can choose these parameters reasonably. The requirement of weight parameter is loose in the segmentation of images that have desired piecewise constant structure. However, the medical imaging and the property of organs will lead to heterogeneity. Moreover, the adjoining tissues have little gray level differences. Therefore, the traditional CV model cannot achieve accurate segmentation and the controlling parameters are difficult to set in medical images.

Consider that when *E*_*in*_(*C*) < *E*_*out*_(*C*), the interior homogeneity is greater than that of the exterior. Thus, enhancing the weight of the exterior homogeneity in the energy function is necessary to reduce the influence of lesser homogeneity to energy. When *E*_*in*_(*C*) = *E*_*out*_(*C*), enhancing the weight of the interior homogeneity is necessary. The energy function will be minimal when the homogeneity of the two regions is balanced.

To address the difficulties mentioned above, this paper utilizes entropy as a weight. The fitting energy is given by

$$F\left(\phi \right)=E_{in}\left(\phi \right){\left|I\left(z\right)-c_{i}\right|}^{2}H\left(\phi \right)+E_{out}\left(\phi \right){\left|I\left(z\right)-c_{o}\right|}^{2}\left(1-H\left(\phi \right)\right)$$ (10)

**Fig 2:**
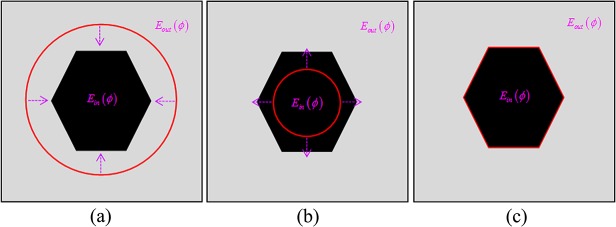
The dominant direction of the weights of the interior and exterior. (a) *E*_*in*_(*ϕ*) > *E*_*out*_(*ϕ*). (b) *E*_*in*_(*ϕ*) < *E*_*out*_(*ϕ*). (c) *E*_*in*_(*ϕ*) = *E*_*out*_(*ϕ*).

In the equation (10), when *E*_*in*_(*ϕ*) < *E*_*out*_(*ϕ*), the weight of the exterior homogeneity is enhanced. When *E*_*in*_(*ϕ*) = *E*_*out*_(*ϕ*), the energy function will acquire the minimum. As entropy changes along with the evolving curve, the weight of the homogeneity over two regions in the energy function will adjust automatically and adaptively. These basic remarks are illustrated in **Fig. 2**, the mauve arrow point to the dominant direction of the weight of interior and the exterior region.

#### 2.5 Adaptive window of local energy of points along the curve

**Fig 3:**
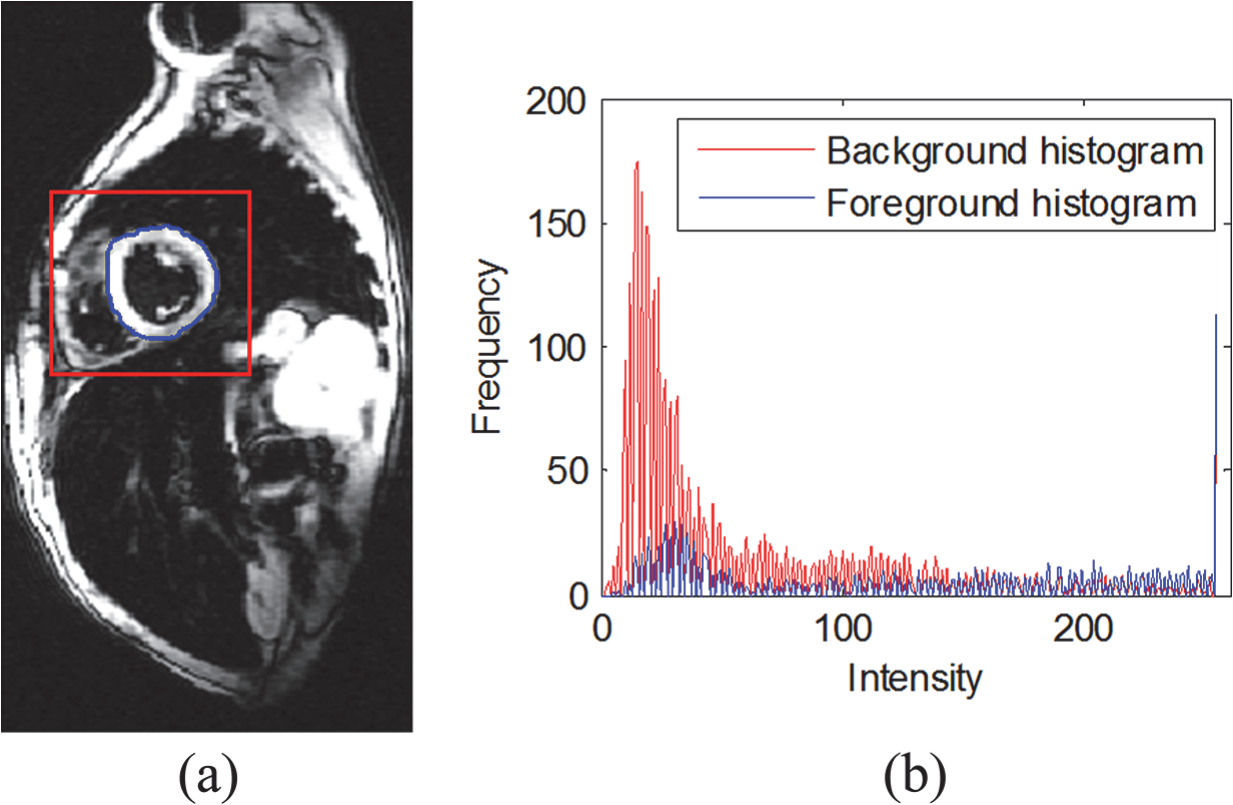
The considerable challenge in medical image segmentation. (a) The background and the foreground in the cardiac MR image. (b) The histograms of the background and the foreground.

In the CV model, the image intensities are assumed to be statistically homogeneous in the foreground and background. However, the assumption does not hold for some general images. Due to image intensity inhomogeneity and complex construction in medical images, the CV and ECV models often fail to achieve accurate segmentation results. **Fig. 3.** illustrates the considerable challenge in medical image segmentation. It is obvious that the image intensity distributions of both the foreground and the background vary sharply; so it is difficult for the CV to find appropriate constants to fit the foreground and the background. Meanwhile, the foreground and background share the similar intensity to some degree. The heterogeneity of regions and the presence of nearby structures of similar image intensity affect the curve evolution, leading to the detection of false boundaries. CV and ECV models are unable to segment the entire object accurately due to the difficulty to find two optimal constants used as the means of the interior and exterior. These models do not include any local neighborhood information which is crucial for segmentation of medical images with image intensity inhomogeneity.

**Fig 4:**
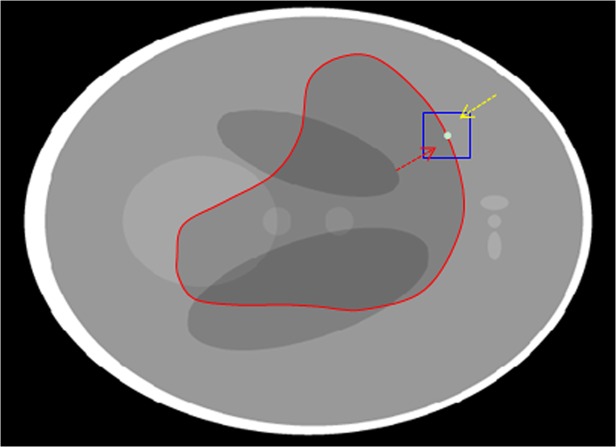
The neighborhood of a point near the curve is split by the evolving curve into the local interior (red arrow) and local exterior (yellow arrow) regions.

To solve the above problems, the information of the local regions inside and outside the curve in neighborhoods of points near the evolving curve [34] can be utilized. The object can be described in terms of smaller homogeneous local regions. In each of this small local region, the image intensities can be assumed to be statistically homogeneous. Let *C* be a closed contour in the image domain Ω, which separates each small region into two regions. As shown in **Fig. 4**, the irregular contour is the evolving curve and the square is the neighborhood of a point near the curve. Arrows point to the local regions inside and outside the contour in the neighborhood. The exterior and interior of the local region can be represented with local statistics.

**Fig 5:**
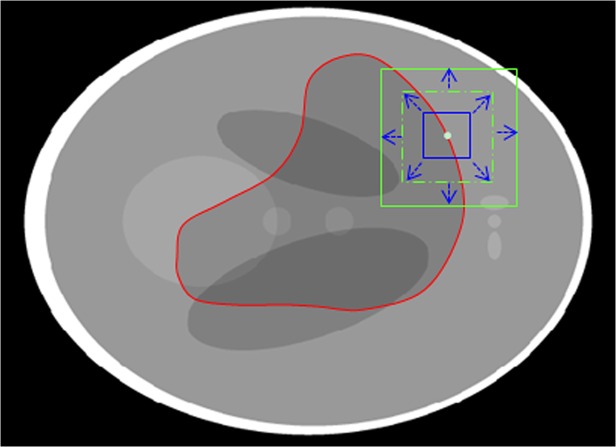
The adaptive neighborhood window. The blue rectangle is the initial and the bigger green rectangle is the adaptive neighborhood window.

Segmentation result is sensitive to the size of the local neighborhood. To avoid the effect of different localization sizes, we use an adaptive window which is estimated by the local entropy of the neighborhood. The evolving curve cannot move to minimize the energy computed in the heterogeneous region. If the entropy of the region is small, this region is homogeneous and we will increase the size of the window until the entropy of the region is more than the threshold. As shown in **Fig. 5**, the blue rectangle is the initial neighborhood window and the bigger green rectangle is the adaptive neighborhood window, which is determined by the entropy of the window. Instead of selecting a fixed size for all the neighborhood windows, we calculate a local window size for each point on the curve adaptively.

The mean of the local interior *u*_*j*_ and local exterior *v*_*j*_, respectively, can be written as

$$u_{j}=\frac{\int_{\Omega_{j}}I\left(x\right)H\left(\phi \left(x\right)\right)dx}{\int_{\Omega_{j}}H\left(\phi \left(x\right)\right)dx},\;v_{j}=\frac{\int_{\Omega_{j}}I\left(x\right)\left(1-H\left(\phi \left(x\right)\right)\right)dx}{\int_{\Omega_{j}}\left(1-H\left(\phi \left(x\right)\right)\right)dx}$$ (11)

where Ω_*j*_ denotes the neighborhood domain of *j*-th point in an image *I*(*x*), and *x* is the spatial variable. The fitting energy of the neighborhood centered on the point *j* is shown as follow:

$$F_{j}\left(\phi \right)=E_{in}^{j}\left(\phi \right){\left|I\left(x\right)-u_{j}\right|}^{2}H\left(\phi \right)+E_{out}^{j}\left(\phi \right){\left|I\left(x\right)-v_{j}\right|}^{2}\left(1-H\left(\phi \right)\right)$$ (12)

Given a center point *j*, the fitting energy *F*_*j*_(*ϕ*) can be minimized when the contour *C* is exactly on the object boundary and the fitting values *u*_*j*_ and *v*_*j*_ optimally approximate the local image intensities on the two sides of *C*. To obtain the entire object and optimize the fitting energy of the image, each point is considered separately and moved to minimize the energy computed in its neighborhood. The fitting energy of the image is the sum of a family of energy computed in the neighborhood of points near the evolving curve and is accomplished by multiplication with the Dirac function *δ*(*ϕ*), as shown in the following equation

$$F\left(\phi \right)=\int_{\Omega }\delta \left(\phi \right)\int_{\Omega }F_{j}\left(\phi \right)dxdz$$ (13)

#### 2.6 Gaussian Regularizing and Partial differential equation

As pointed out by Shi et al. [35], the evolution of a function according to its Laplacian is equivalent to Gaussian filtering the initial condition of the function. So the previous iteration result of the level set function can be viewed as the initial condition for the next iteration:

$$\phi^{n+1}=G_{\sqrt{\text{Δ}t}}*\phi^{n}=G_{\xi }*\phi^{n}$$ (14)

In order to enhance the smoothing capacity, we choose the *ξ* larger than the square root of the time-step Δ*t*. As we use a Gaussian kernel to regularize the level set function the traditional regularized term div(∇*ϕ*/|∇*ϕ*|)*δ*(*ϕ*) can be re-moved [36].

The final energy function of the adaptive Gaussian regularizing CV model using entropy and LNI is:

$$\varepsilon \left(\phi \right)=\nu \int_{\Omega }H\left(\phi \right)dz+\int_{\Omega }\delta \left(\phi \right)\int_{\Omega }F_{j}\left(\phi \right)dxdz$$ (15)

and the corresponding curve evolving equation is:

$$\frac{\partial \phi \left(z\right)}{\partial t}=\delta \left(\phi \left(z\right)\right)\left[-\nu -\int_{\Omega }\nabla_{\phi }F_{j}\left(\phi \left(x\right)\right)dx\right]$$ (16)

where $\nabla_{\phi }F_{j}\left(\phi \right)=\delta \left(\phi \right)(E_{in}^{j}\left(\phi \right){\left|I\left(x\right)-u_{j}\right|}^{2}-E_{out}^{j}\left(\phi \right){\left|I\left(x\right)-v_{j}\right|}^{2})$.

## Results and Discussions

In this section, we evaluate and compare the proposed model (SMLV) with the CV model, the LBF model [21], and recently developed local neighborhood region-based CV models (NCV) [22] using both synthetic and in vivo medical images. The results of these experiments demonstrate the desirable properties of the new model. All the experiments are conducted in a computer with an Intel Core Duo processor, 3.10 GHz, and 4 GB RAM. In the NCV model, we choose a fixed and predetermined neighborhood size *n* × *n* of an image size *M* × *N*, where the optimal size is calculated as *n* = (*M* + *N*)/16 according to our previous studies.

### 1. Analyzing the entropy-weighted CV

**Fig 6:**
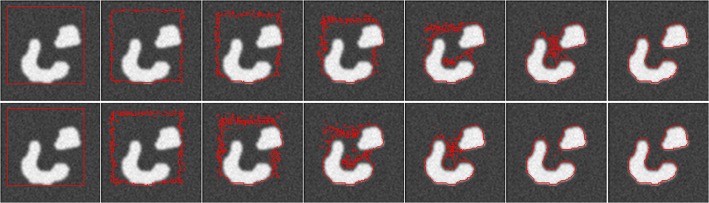
The segmentations of CV model (Row 1) and entropy-weighted model (Row 2). Column 1: the initializations. Column 2, 3, 5 and 6 shows the evolution process for iterations 50, 100, 150, 200, and 250 respectively. Column 7: the final segmentations of CV (iterations 320) and entropy-weighted model (iterations 270).

**Fig 7:**
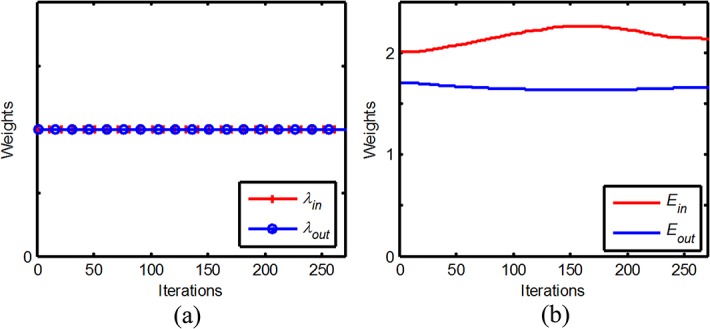
The weights of the interior and exterior. (a) CV model. (b) The entropy-weighted CV model.

The first experiment is designed to evaluate the entropy-weighted CV model, which is used to segment the synthetic image, a typical example of image with high contrast (**Fig. 6**). To show the effect of the entropy, we set *μ* = 0.01 × 255^2^, *ν* = 0, *λ*_*in*_ = 1 and *λ*_*out*_ = 1 in CV model, and *μ* = 0.01 × 255^2^, *ν* = 0 in entropy-weighted CV model. From the segmentation process shown in **Fig. 6**, we can see that the entropy-weighted CV model can segment the object with less iteration compared with CV model. To further study the behavior of entropy, the **Fig. 7** shows the entropy can adaptively adjust the proportion of the interior and exterior of the curve

### 2. Segmentation of LV contours(the endocardium and epicardium of the left ventricle)

In this section, we validated the proposed model using a synthetic image and mass CMR images for the LV endocardium and epicardium segmentation. The myocardium and background area are inhomogeneous. LVs and adjoining organs overlap in some images presented. In addition, some areas in LV regions have similar gray level to surrounding tissues. These results partly explain why the CV model results in poor LV segmentation.

**Fig 8:**
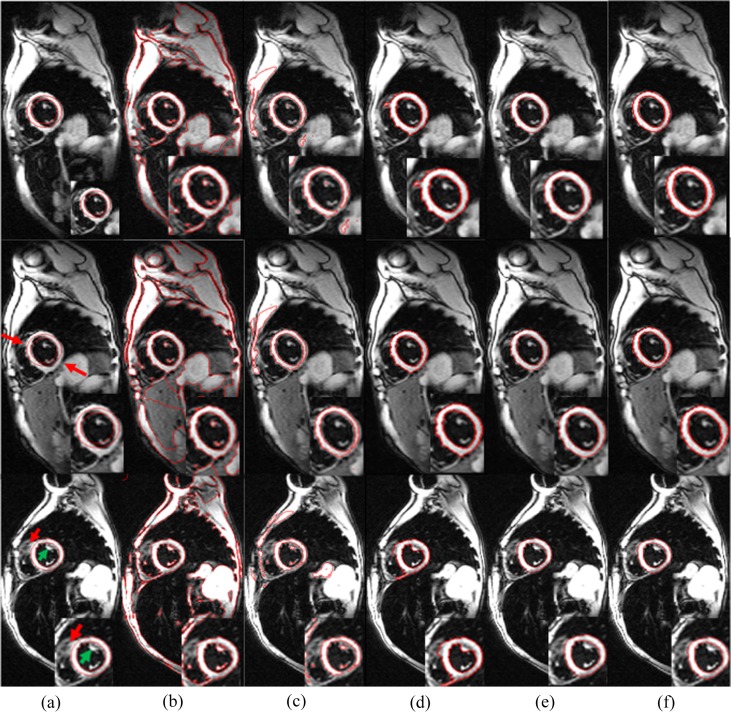
Comparing segmentations. (a) Initial contour set by the CHT. (b) LBF. (c) CV. (d) NCV. (e) SMLV. (f) Ground truth.

**Fig. 8** shows the results of automatic segmentation using the four models (LBF, CV, NCV and the present method), compared with the reference manual segmentation (as Ground Truth) by medical experts. The first column shows three cardiac MR images and their initializations, which are detected through the CHT, from the second to the fifth columns show results for the automatic segmentation using the four models, and the last column presents the manual segmentation. From **Fig. 8**, the boundaries of the LVs were automatically detected accurately by the present model proposed in this paper, which exhibited better results than the other two models. The NCV model got some wrong boundaries in the walls of the LV areas, and the high-intensity regions were considered as objects. On the other hand, the LBF and CV model may detect some wrong contours to some extent. Hence, **Fig. 8** shows the various advantages of the present model in medical image segmentation. Table 1 lists the controlling parameters of the four methods. It is obvious that the present method not only avoids to the optimal configuration of controlling parameters due to the action of entropy, but also takes shortest time to achieve accurate segmentations.

**Table 1:** The controlling parameters of the CV, LBF, NCV models and the present method.

Method	Patient	*λ*_*in*_	*λ*_*out*_	*μ*	*ν*
	1	1	1	0.5 × 255^2^	0.2
CV	2	1	1	0.5 × 255^2^	0.2
	3	1	1	0.5 × 255^2^	0.2
	1	1	1	0.03 × 255^2^	0.2
LBF	2	1	1	0.03 × 255^2^	0.2
	3	1	1	0.05 × 255^2^	0.2
	1	1	1	0.1	0.2
NCV	2	1	1	0.2	0.1
	3	1	1	0.2	0.15
	1	—	—	—	0.05
SMLV	2	—	—	—	0.05
	3	—	—	—	0.05

**Fig 9:**
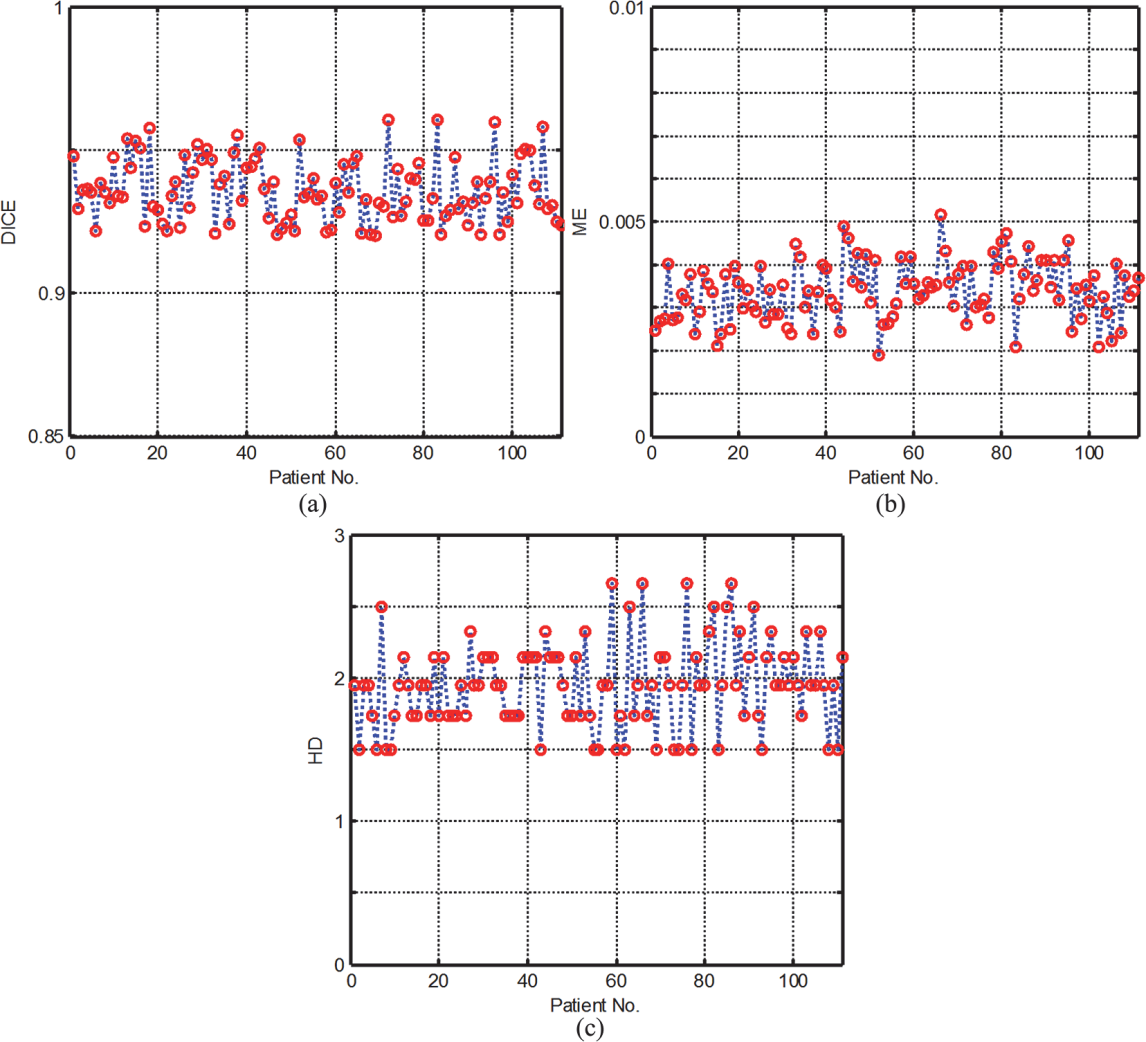
The Dice, ME and HD values for the 111 patients segmented by SMLV method.

**Fig 10:**
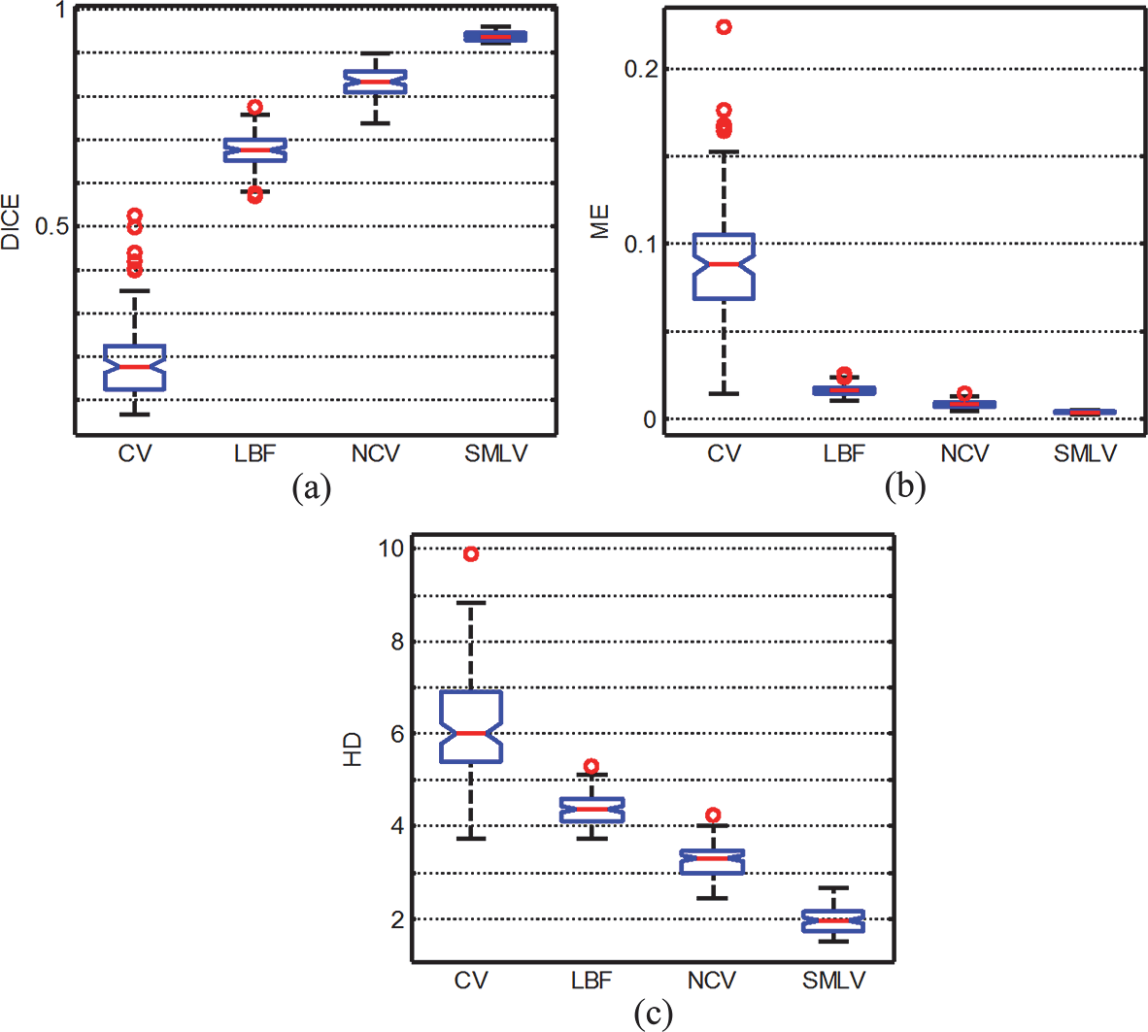
Boxplot representations of Dice, ME and HD values for the 111 patients segmented by various methods.

For quantitative evaluation among the four automatic segmentation methods, we utilize the DICE [37], ME [38] and Hausdorff distance (HD) [39] to test the various algorithms, as follows

$$\text{DICE}=\frac{2\times \left|F_{Manual}\cap F_{Auto}\right|}{\left|F_{Manual}\right|+\left|F_{Auto}\right|}$$ (17)

$$\text{ME=}1-\frac{\left|B_{Manual}\cap B_{Auto}\right|+\left|F_{Manual}\cap F_{Auto}\right|}{\left|B_{Manual}\right|+\left|F_{Manual}\right|}$$ (18)

$$HD\left(F_{Manual},F_{Auto}\right)=\max\left[h\left(F_{Manual},F_{Auto}\right),h\left(F_{Auto},F_{Manual}\right)\right]$$ (19)

where *F*_*Manual*_ and *B*_*Manual*_ are the foreground and background of the manual algorithm respectively, as the *F*_*Auto*_ and *B*_*Auto*_ are foreground and background of the automatic algorithm. The |*F*_*Manual*_| denotes the number of pixels assigned to the foreground by the medical expert. The higher the value of DICE, the better the overall performance of the segmentation will be. DICE Index equal to 1 suggests a good match between manual and automatic segmentation. The lower the ME ratio, the fewer the misclassified pixels are. HD measures the maximum distance between two contours. **Fig. 9** illustrates the DICE, HD and ME values of the SMLV method. The DICE values are ranged from 0.9203 to 0.9607, the ME 0.0019 to 0.0052 and the HD from 1.5000 to 1.6623. To compare the automatic segmentation of the LV, we show in Fig. 10 the Dice values, scaled to a range of the same size (0.35). A quick look at the Dice similarity measures, ME and HD can be done also using a boxplot, see **Fig. 10A-C**. Note that there is substantially less variability in the ratings of our method than others. According to **Fig. 10A-C**, it can be observed that the myocardium extraction by the SMLV is most accurate with least misclassified pixels. The segmentation results demonstrate that the present approach outperforms the other traditional methods and has good agreement with the manual method.

### 3. Computation time

**Fig 11:**
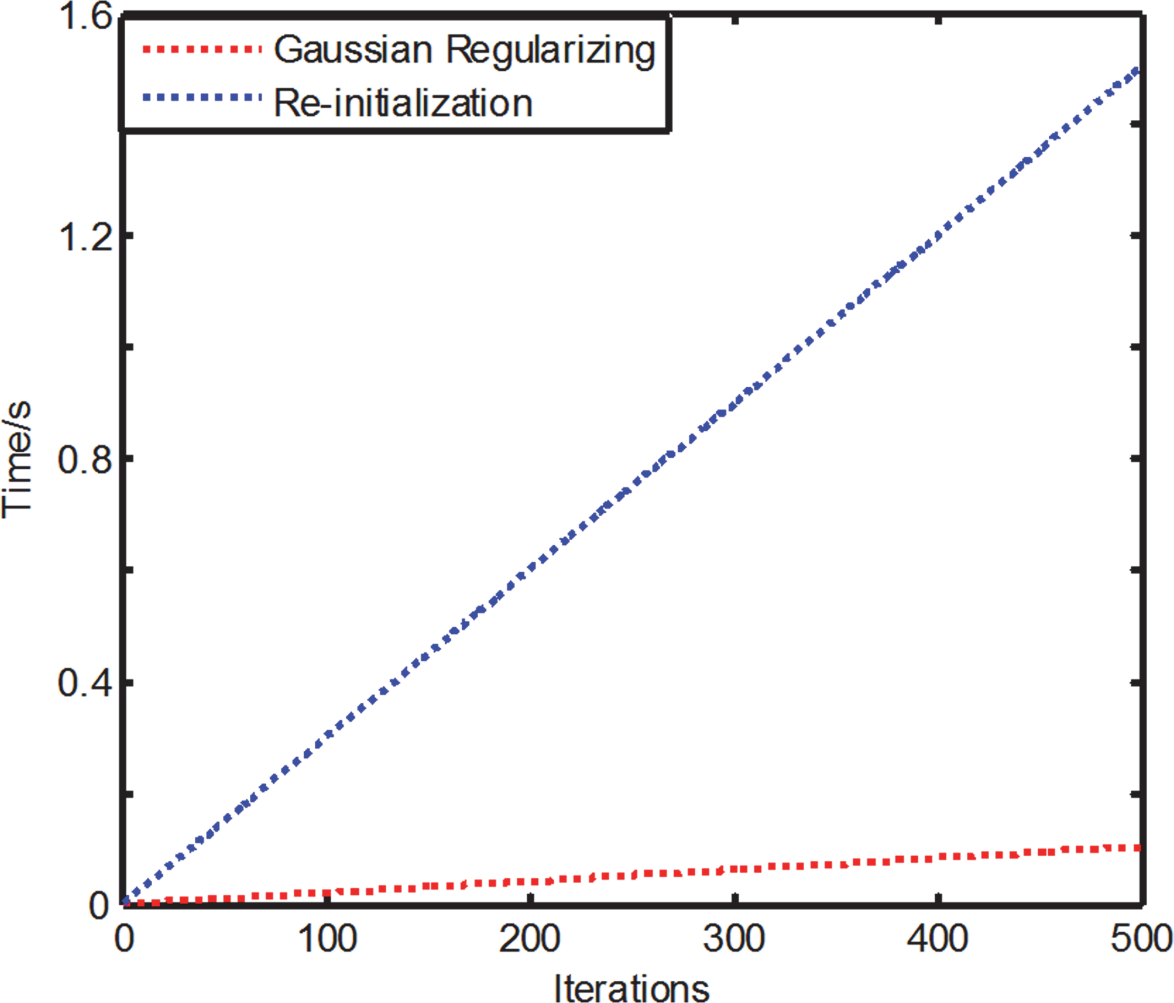
Comparing the Gaussian regularizing with re-initialization level set function.

**Fig. 11** shows the comparing the time efficiency of the Gaussian regularizing with the re-initialization level set function. The Gaussian regularizing removes re-initialization and the traditional regularized term, so is capable of keeping the curve evolution stable and smooth while reducing the computation time of the algorithm.

**Fig 12:**
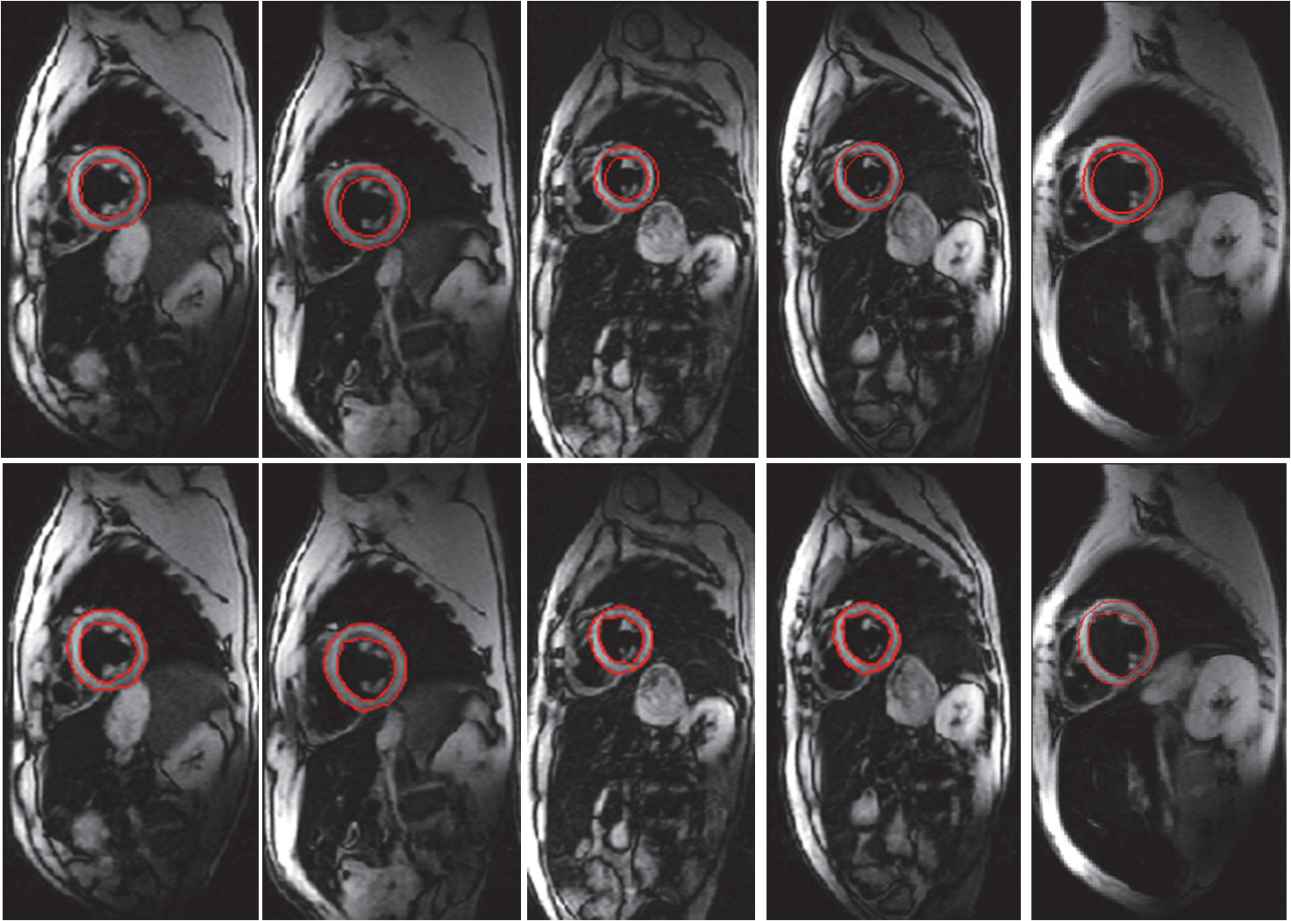
The other patients’ segmentations by the present method (10 iterations). Row 1: the initial contours set by the CHT. Row 2: the segmentation endocardium and epicardium of the left ventricle.

In **Fig. 12**, we show more examples of accurate segmentations by the present method.

## Conclusions

We have presented an automatically method for myocardium segmentation from black-blood cardiac images. By utilizing the CHT, the present method can detect the LV endocardium and epicardium automatically. Traditional CV model is unsuitable for medical segmentation as it has limitation in parameter setting and suffers from heterogeneity intensities. To address the first issue, we utilize entropy to weight the energies of inside and outside the contour. For the second one, we incorporate the local neighborhood information to reduce the effect of inhomogeneity inside regions. Instead of selecting a fixed neighborhood window in NCV, we calculated the local window size for each point on the curve adaptively to reduce the model sensitivity to initialization. In addition, we use the Gaussian to regularize the level set function for removing traditional regularized term and the re-initialization, which not only ensure the smoothness and stability of the level set function, but also reduce the computation time. Encouraging experimental results on the real medical images demonstrated that the proposed algorithm is very robust, efficient and much less sensitive to the initial contour.

The limitation of this method is that the CHT may be failed in some images with the bright region of the fat and muscle. In the future, we will try to preproccess these images for improving the robust of the initialization by the CHT. MRI-T2* has been accepted as a clinical tool for monitoring iron overload in thalassemia patients. Our future studies will focus on the assessing the myocardial iron loading in CMR images. Due to the universality of bright-blood images, the present method will be improved in order to segment these bright-blood images for T2* assessment.

## References

[1] Organization WH (2007) Cardiovascular disease fact sheet no. 317. February 2007.

[2] Anderson L, Holden S, Davis B, Prescott E, Charrier C, Bunce N, et al (2001) Cardiovascular T2-star (T2*) magnetic resonance for the early diagnosis of myocardial iron overload. Eur Heart J 22: 2171–2179. 11913479 10.1053/euhj.2001.2822

[3] Kanungo T, Mount DM, Netanyahu NS, Piatko CD, Silverman R, Wu AY (2002) An efficient k-means clustering algorithm: Analysis and implementation. IEEE Trans Pattern Anal Mach Intell 24: 881–892.

[4] Gath I, Geva AB (1989) Unsupervised optimal fuzzy clustering. IEEE Trans Pattern Anal Mach Intell 11: 773–780.

[5] Rohlfing T, Russakoff DB, Maurer CR (2004) Performance-based classifier combination in atlas-based image segmentation using expectation-maximization parameter estimation. IEEE Trans Med Imaging 23: 983–994. 15338732 10.1109/TMI.2004.830803

[6] Schillo C, Herrmann G, Ackermann F, Posch S, Sagerer G (1995) Statistical classification and segmentation of biomolecular surfaces. IEEE International Conference on Image Processing. pp. 560–563.

[7] Singh V, Marinescu DC, Baker TS (2004) Image segmentation for automatic particle identification in electron micrographs based on hidden markov random field models and expectation maximization. J Struct Biol 145: 123–141. 15065680 10.1016/j.jsb.2003.11.028 PMC4167639

[8] Woods JW, Dravida S, Mediavilla R (1987) Image estimation using doubly stochastic Gaussian random field models. IEEE Trans Pattern Anal Mach Intell: 245–253. 10.1109/tpami.1987.4767898 21869394

[9] Kichenassamy S, Kumar A, Olver P, Tannenbaum A, Yezzi A Jr (1996) Conformal curvature flows: from phase transitions to active vision. Arch Ration Mech An 134: 275–301.

[10] Goldenberg R, Kimmel R, Rivlin E, Rudzsky M (2001) Fast geodesic active contours. IEEE Trans Image Process 10: 1467–1475. 10.1109/83.951533 18255491

[11] Kass M, Witkin A, Terzopoulos D (1988) Snakes: Active contour models. Int J Comput Vision 1: 321–331.

[12] Mumford D, Shah J (1989) Optimal approximations by piecewise smooth functions and associated variational problems. Commun Pur Appl Math 42: 577–685.

[13] Chan TF, Vese LA (2001) Active contours without edges. IEEE Trans Image Process 10: 266–277. 10.1109/83.902291 18249617

[14] Lankton S, Nain D, Yezzi A, Tannenbaum A. Hybrid geodesic region-based curve evolutions for image segmentation; 2007 International Society for Optics and Photonics pp. 65104U-65104U-65110.

[15] Besag J (1986) On the statistical analysis of dirty pictures. Journal of the Royal Statistical Society Series B (Methodological): 259–302.

[16] Černý V (1985) Thermodynamical approach to the traveling salesman problem: An efficient simulation algorithm. J Optimiz Theory App 45: 41–51.

[17] Zhang T, Freedman D (2003) Tracking objects using density matching and shape priors. IEEE International Conference on Computer Vision. pp. 1056–1062.

[18] Paragios N, Chen Y, Faugeras OD (2010) Handbook of mathematical models in computer vision: Springer Publishing Company, Incorporated.

[19] Sum K, Cheung PY (2006) A novel active contour model using local and global statistics for vessel extraction. 28th Annual International Conference of the IEEE. pp. 3126–3129. 10.1109/IEMBS.2006.260817 17946550

[20] Sum K, Cheung PY (2008) Vessel extraction under non-uniform illumination: a level set approach. IEEE Trans Bio-med Eng 55: 358–360. 10.1109/TBME.2007.896587 18232383

[21] Li C, Kao C-Y, Gore JC, Ding Z (2007) Implicit active contours driven by local binary fitting energy. IEEE Conference on computer vision and pattern recognition. pp. 1–7.

[22] Lankton S, Tannenbaum A (2008) Localizing region-based active contours. IEEE Trans Image Process 17: 2029–2039. 10.1109/TIP.2008.2004611 18854247 PMC2796112

[23] Wang XF, Huang DS, Xu H (2010) An efficient local Chan-Vese model for image segmentation. Pattern Recogn 43: 603–618.

[24] Smith GC, Carpenter JP, He T, Alam MH, Firmin DN, Pennell DJ (2011) Value of black blood T2* cardiovascular magnetic resonance. J Cardiovasc Magn Reson 13: 21 10.1186/1532-429X-13-21 21401929 PMC3062187

[25] Cocosco CA, Niessen WJ, Netsch T, Vonken EJPA, Lund G, Stork A, et al (2008) Automatic image-driven segmentation of the ventricles in cardiac cine MRI. J Magn Reson Imaging 28: 366–374. 10.1002/jmri.21451 18666158

[26] Huang S, Liu J, Lee L, Venkatesh S, Teo L, Au C, et al. (2009) Segmentation of the left ventricle from cine MR images using a comprehensive approach. MIDAS J-Card MR Left Ventricle Segmentation Challenge. 10.1007/s10278-010-9315-4 PMC3138938 20623156

[27] Jolly M (2009) Fully automatic left ventricle segmentation in cardiac cine MR images using registration and minimum surfaces. The MIDAS Journal-Cardiac MR Left Ventricle Segmentation Challenge 4. 10.1007/978-3-642-04271-3_110 20426198

[28] Zheng Q, Lu Z, Zhang M, Feng Q, Chen W. Gaussian Regularizing CV Model Using Entropy and Neighborhood Information; 2013; New York Springer pp. 1832–1835.

[29] Zheng Q, Feng Y, Wei X, Feng M, Chen W, Lu Z, et al. (2014) Automated interventricular septum segmentation for black-blood myocardial T2* measurement in thalassemia. J Magn Reson Imaging. 10.1002/jmri.24662 24862942

[30] Kimme C, Ballard D, Sklansky J (1975) Finding circles by an array of accumulators. Commun Acm 18: 120–122.

[31] Osher S, Sethian JA (1988) Fronts propagating with curvature-dependent speed: algorithms based on Hamilton-Jacobi formulations. J Comput Phys 79: 12–49.

[32] Portes de Albuquerque M, Esquef I, Gesualdi Mello A (2004) Image thresholding using Tsallis entropy. Pattern Recogn Lett 25: 1059–1065.

[33] Diniz P, Murta-Junior L, Brum D, de Araújo D, Santos A (2010) Brain tissue segmentation using q-entropy in multiple sclerosis magnetic resonance images. Braz J Med Biol Res 43: 77–84. 19936540 10.1590/s0100-879x2009007500019

[34] Luo X-p, Tian J, Lin Y (2002) An algorithm for segmentation of medical image series based on active contour model. Journal of Software 13: 1050–1058. 12435196

[35] Shi Y, Karl WC. Real-time tracking using level sets; 2005 IEEE pp. 34–41.

[36] Zhang K, Song H, Zhang L (2010) Active contours driven by local image fitting energy. Pattern Recogn 43: 1199–1206.

[37] Mayer A, Greenspan H (2009) An adaptive mean-shift framework for MRI brain segmentation. IEEE Trans Med Imaging 28: 1238–1250. 10.1109/TMI.2009.2013850 19211339

[38] Sezgin M (2004) Survey over image thresholding techniques and quantitative performance evaluation. J Electron Imaging 13: 146–168.

[39] Alt H, Guibas LJ (1999) Discrete geometric shapes: Matching, interpolation, and approximation. Handbook of computational geometry 1: 121–153.

